# Supplementary material for: Photodegradation of carbon dots cause cytotoxicity
Source: Nat Commun. 2021 Feb 5;12:812. doi: 10.1038/s41467-021-21080-z (PMC7864953; doi:10.1038/s41467-021-21080-z)
Supplement: Supplementary file 1 — Supplementary Information [file 41467_2021_21080_MOESM1_ESM.pdf]

Supplementary Information

Photodegradation of carbon dots cause cytotoxicity

Yue-Yue Liu[1#], Nan-Yang Yu[1#], Wen-Di Fang[1#], Qiao-Guo Tan[2], Rong Ji[1], Liu-Yan Yang[1], Si Wei[1]*, Xiao-Wei Zhang[1]* and Ai-Jun Miao[1]*

[1]State Key Laboratory of Pollution Control and Resource Reuse, School of the Environment, Nanjing University, Nanjing, Jiangsu Province, 210046, China.

[2]Key Laboratory of the Coastal and Wetland Ecosystems of Ministry of Education, College of the Environment and Ecology, Xiamen University, Xiamen, Fujian Province, 361102, China.

[#]These authors contributed equally.

*Corresponding authors:

S. Wei, School of the Environment, Nanjing University, Mail box 24, Xianlin Road 163, Nanjing 210046, Jiangsu Province, China PRC (mailing address), 86-25-89680356 (phone), weisi@nju.edu.cn (email)

X.-W. Zhang, School of the Environment, Nanjing University, Mail box 24, Xianlin Road 163, Nanjing 210046, Jiangsu Province, China PRC (mailing address), 86-25-89680623 (phone), zhangxw@nju.edu.cn (email)

A.-J. Miao, School of the Environment, Nanjing University, Mail box 24, Xianlin Road 163, Nanjing 210046, Jiangsu Province, China PRC (mailing address), 86-25-89680255 (phone), miaoaj@nju.edu.cn (email)

Cell invasion and transformation assays: HepG2 cells were seeded at a density of 1×10^5 cells/well in 12-well plates (Thermo Fisher Scientific, MA, USA). They were then exposed to three 5-fold dilutions of non-irradiated carbon dots (n-CD, 12–300 mg carbon/L), 8-day irradiated CDs (i_8-CD, 0.08–2 mg carbon/L) as well as the < 3 kD (i_8-CD$_{<3kD}$, 0.12–3 mg carbon/L) and > 3 kD fractions (i_8-CD$_{>3kD}$, 12–300 mg carbon/L) of i_8-CD for 24 h. The exposure concentrations were the same as the three highest concentrations used in the RHT experiment for n-CD, i_8-CD, i_8-CD$_{<3kD}$, and i_8-CD$_{>3kD}$, respectively. Two control treatments without any addition of CDs (control) or in the presence of 4 μM benzopyrene with well-known carcinogenic effects (positive control) were also included. Afterward, the cells were collected for the invasion and transformation assays below.

Cell invasion ability was investigated through the quantitative CytoSelect™ 96-well Cell Invasion Assay Kit (#CBA-112, Cell Biolabs, INC., CA, USA), following the manufacturer's instructions. Briefly, the CD-exposed HepG2 cells (1×10^5 cells per well) were first seeded into a serum-free medium of the upper chamber, which was separated from the lower chamber by a basement membrane layer at the bottom. The HepG2 cells in upper chamber were allowed to invade toward the 10% FBS in the lower chamber for 24 h. Those that were able to degrade the matrix proteins in the layer and ultimately pass through the membrane were stained with CyQuant® GR Dye. The fluorescence intensity (480 nm/520 nm) was measured using a Synergy H4 Hybrid microplate reader (BioTek Instruments, Inc., VT, USA).

Cell transformation ability was examined by the CytoSelect™ 96-well Cell Transformation Assay Kit (Cell Biolabs, #CBA-130). Briefly, a base agar layer was prepared

by mixing equal volumes of 1.2% agar solution and 2× DMEM/20% FBS medium in each well of a 96-well flat-bottom microplate. The CD-exposed HepG2 cells (2,000 cells per well) were seeded in a top layer by mixing equal volumes of the cell suspension, 1.2% agar solution, and 2×DMEM/20% FBS (1:1:1), and incubated for 6 days after covering the solidified cell agar layer with 100 μl of DMEM medium. Then 1× matrix solubilization solution was applied to solubilize the agar matrix completely and the cell growth was determined through the CyQuant® GR Dye (485 nm/520 nm).

Fractionation of the degradation products and cell viability assay: The photodegradation products as enriched by solid-phase extraction were fractionated through an XBridge Prep C18 column (10 mm × 100 mm, 5 μm) by preparative high-performance liquid chromatography (HPLC) (Waters AutoPurification, Waters, USA). The mobile phase was water (LC-MS grade) (A) and methanol (B) with a flow rate of 5 mL/min. The HPLC gradient was as follows: 95% solvent A for 3.5 min, decreased to 0% solvent A until 49.5 min and held until 65 min, returned to 95% solvent A until 65.5 min and held at 95% solvent A until 72 min. Nine fractions were obtained with the collection time 8 min for each fraction. The photodegradation products in each fraction was then concentrated by solid-phase extraction. The chemical components in each fraction was further screened by HPLC-QTOF and compared with what was observed by the identification experiment. The cytotoxicity of each fraction was also examined with HepG2 cells following the same procedure as the other cell viability experiments.

Table 1. Concentration-effect models used to fit the fold changes in the expression of genes identified in the reduced human transcriptome analysis.

Curve type	Model name	Equation	Parameter
Sigmoid	Three-parameter log-logistic	$y = \frac{d}{1 + \exp(b(\log(x) - \log(e)))}$	*d*=upper limit; *e*=inflection point; *b*=slope
	Four-parameter log-logistic	$y = c + \frac{d - c}{1 + \exp(b(\log(x) - \log(e)))}$	*c*=lower limit; *d*=upper limit; *e* = inflection point; *b* = slope
	Michaelis-Menten	$y = c + \frac{d - c}{1 + e/x}$	*c*=lower limit; *d*=upper limit; *e*=dose yielding a response halfway between c and d
	Weibull I	$y = c + (d - c)\exp(-\exp(b(\log(x) - \log(e))))$	*c*=lower limit; *d*=upper limit; *e*=inflection point; *b*=slope
	Weibull II	$y = c + (d - c)(1 - \exp(-\exp(b(\log(x) - \log(e)))))$	*c*=lower limit; *d*=upper limit; *e*=inflection point; *b*=slope
Linear	Linear	$y = E_0 + \delta x$	E_0=intercept; δ=slope
	Linear-log	$y = E_0 + \delta\log(x + \text{off})$	E_0=intercept; δ=slope; off=a fixed offset parameter
U-shaped	Gaussian	$y = c + (d - c)\exp\left(-0.5\left(\frac{\log(x) - \log(e)}{b}\right)^2\right)$	*c*=back ground effect; *d*=peak effect; *e*=peak position; *b*=width
	Gaussian-log	$y = c(d - c)\exp\left(-0.5\left(\frac{\mathrm{x} - \mathrm{e}}{b}\right)^2\right)$	*c*=back ground effect; *d*=peak effect; *e*=peak position; *b*=width

y = response; *x* = concentration

Table 2. Structures of the 10 chemicals that affected > 50% of the differentially expressed genes (DEGs), Gene Ontology (GO) and Kyoto Encyclopedia of Genes and Genomes (KEGG) pathways co-detected in HepG2 cells exposed to the total unfiltered suspension of 8-day irradiated CDs (i_8-CD) and its < 3 kD fraction containing photolyzed molecules (i_8-CD$_{<3kD}$).

Name	Structure	Name	Structure
cyclosporine	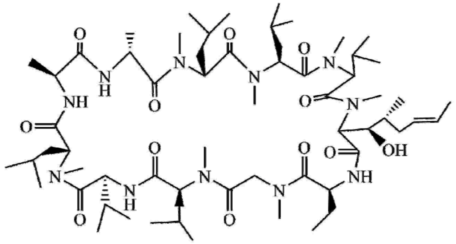	doxorubicin	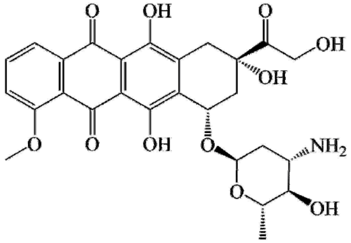
cisplatin	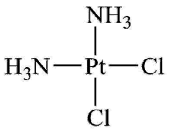	valproic acid	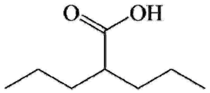
acetaminophen	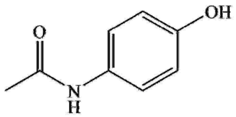	trichostatin	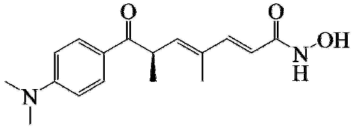
benzo(a)pyrene	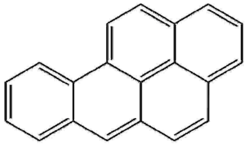	aflatoxin B1	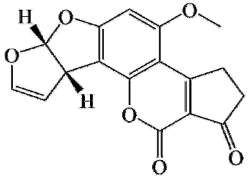
bisphenol A	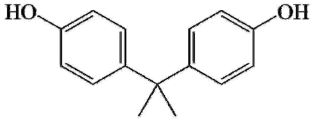	formaldehyde	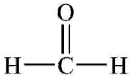

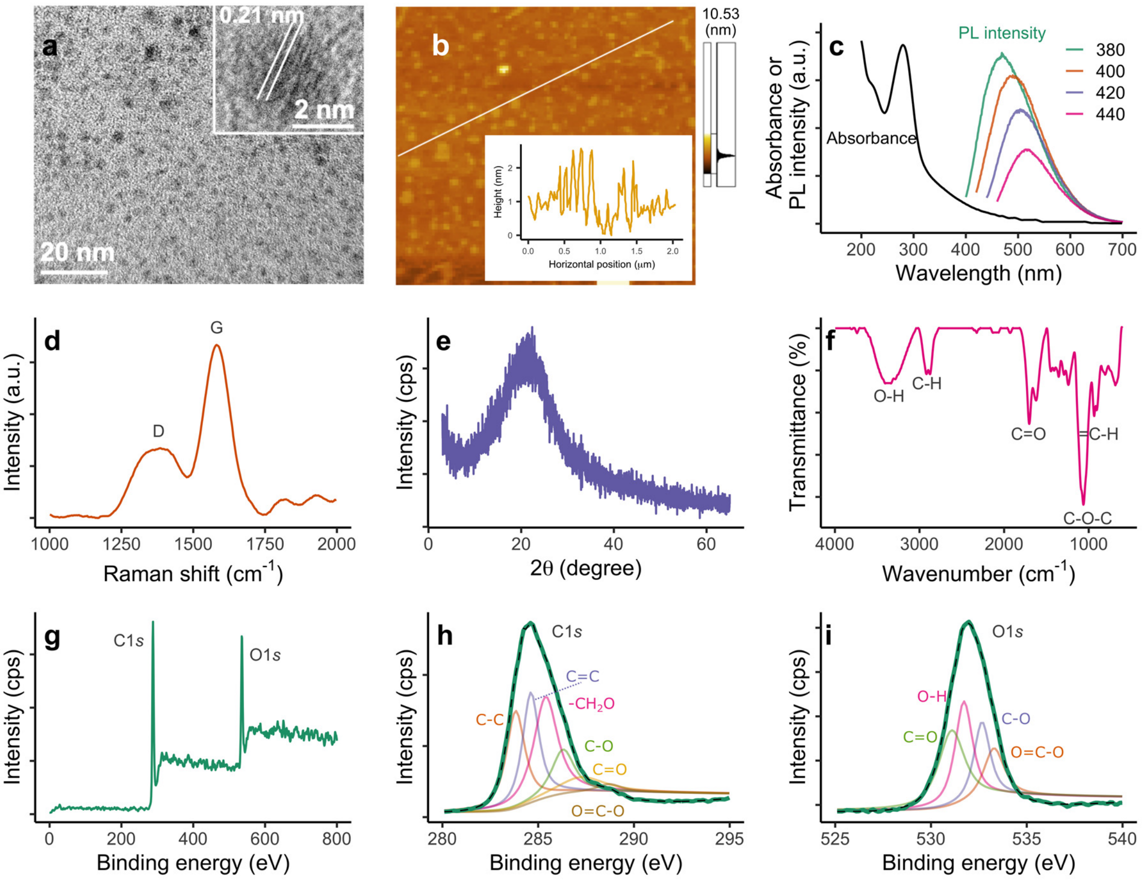

Fig. 1 | Physicochemical characterization of laboratory-synthesized carbon dots (CDs). a, Transmission electron microscopy (TEM) image shows CDs' average size is 3.0 nm. Inset: high-resolution TEM image of a CD with lattice spacing of 0.21 nm. **b**, Atomic force microscopy (AFM) image shows CDs' average size is 2.5 nm. **c,** Two absorption peaks at 224 nm and 280 nm are seen in the UV-Vis spectrum (black curve). As excitation wavelength increased from 380 nm to 440 nm (in 20 nm increments), photoluminescence (PL) emission was red shifted from 469 nm to 516 nm. **d,** Raman spectrum of CDs showing the D and G band. I_D/I_G is 0.4, indicating CDs have greater structural defects than graphite. **e,** Broad peak at 21.4° in the X-ray diffraction spectrum confirms the structural disorder in the CDs. **f-g,** Fourier transform infrared (**f**) and X-ray photoelectron spectra (**g**) along with the deconvoluted C1*s* (**h**) and O1*s* (**i**) peaks reveal hydrophilic groups are present on the CD surface. Images of more than 1000 particles of CDs were taken by TEM or AFM. Source data are provided as a Source Data file.

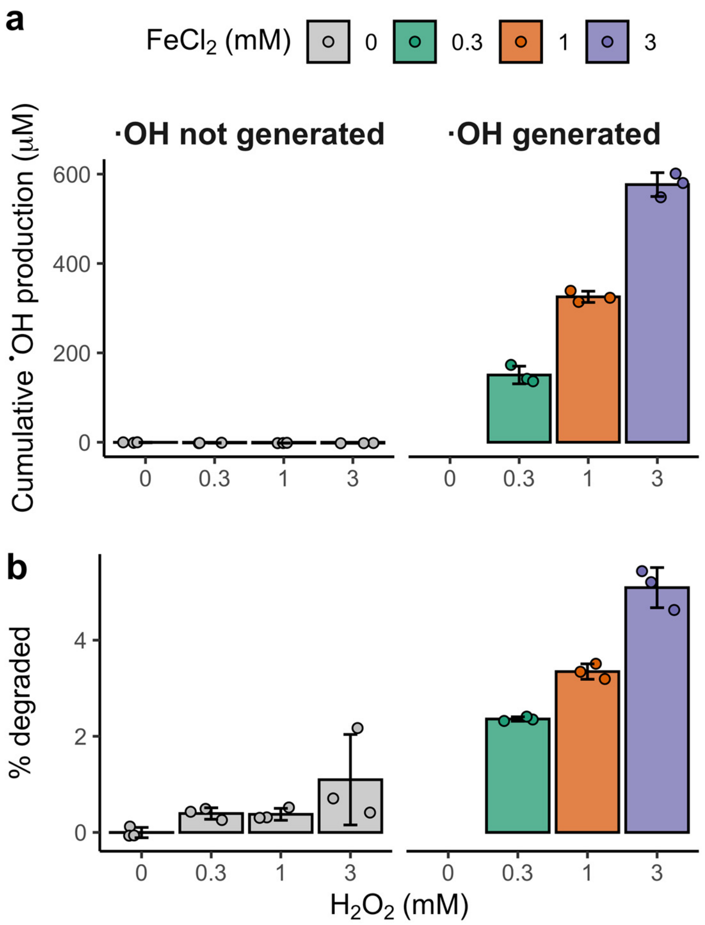

Fig. 2 | Effects of hydroxyl radicals (·OH) on carbon dot (CD) degradation in the dark. a, When 0.3–3 mM $FeCl_2$ was added to the experimental medium, a substantial amount of ·OH was generated, which increased with the increase in the concentration of H_2O_2 and $FeCl_2$. **b,** Following a similar trend as that of ·OH, more CDs degraded in the presence of $FeCl_2$ as compared to the respective control treatments with the same concentration of H_2O_2 but without any addition of $FeCl_2$. Further, more CDs degraded at higher concentrations of H_2O_2 and $FeCl_2$. The positive correlation between ·OH production and CD degradation indicates the important role of ·OH in CD degradation. Data are presented as the mean ± s.d. (n = 3 independent experiments). Source data are provided as a Source Data file.

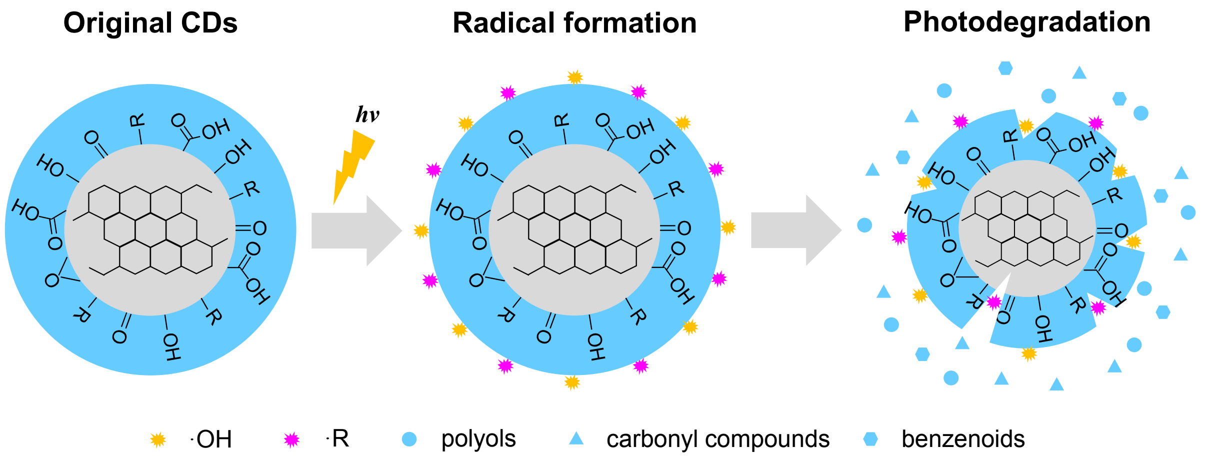

Fig. 3 | Scheme of carbon dot (CD) photodegradation. Upon light irradiation, photogeneration of electrons and holes would occur on the surface of the CDs, resulting in the formation of hydroxyl (·OH) and alkyl (·R) radicals. Conjugated π-bond, surface defects, and functional groups of the CDs were also possibly involved in the generation of radicals. The radicals could then attack the CDs in multiple ways (e.g., transferring electrons to CDs, abstracting H-atom from CDs, hydroxylating CDs by attacking the C=C bonds of CDs) and the CDs thus degraded into molecules containing hydroxyl (polyols), carbonyl, and benzene (benzenoids) groups.

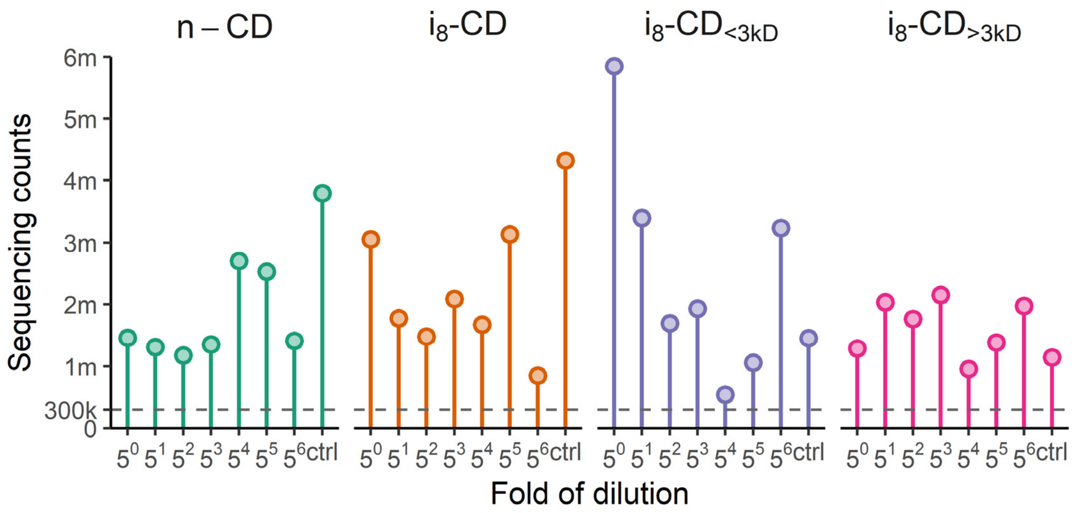

Fig. 4 | Sequencing counts of HepG2 cells exposed to laboratory-synthesized carbon dots (CDs). Four samples [i.e., non-irradiated CDs (n-CD), the total unfiltered suspension of 8-day irradiated CDs (i_8-CD) and its < 3 kD fraction containing photolyzed molecules (i_8-CD$_{<3kD}$) and the > 3 kD fraction containing degraded CDs (i_8-CD$_{>3kD}$)] were included. Each sample contains one vehicle control (0.1% v/v of methanol) and seven 5-fold dilutions of the original sample (i.e., 300 mg carbon/L of n-CD, 2 mg carbon/L of i_8-CD, 3 mg carbon/L of i_8-CD$_{<3kD}$, and 300 mg carbon/L of i_8-CD$_{>3kD}$). Sequence counts ranging from 543,084 to 5,848,694 were obtained. All sequence depths were > 300,000 reads, a level sufficient to detect the signals (counts of > 5) of at least 750 genes, as calculated by Monte Carlo simulations. Source data are provided as a Source Data file.

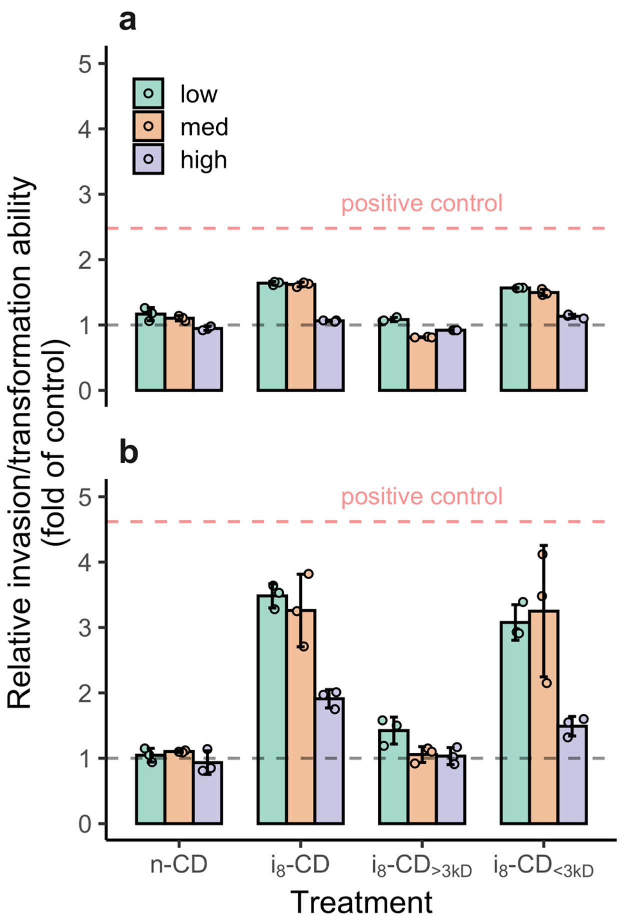

Fig. 5 | Invasion and transformation of HepG2 cells exposed to laboratory-synthesized carbon dots (CDs). CDs [i$_8$-CD, 0.08 (low), 0.4 (med), and 2 (high) mg carbon/L] together with their photolyzed products in the < 3 kD fraction [i$_8$-CD$_{<3kD}$, 0.12 (low), 0.6 (med), and 3 (high) mg carbon/L] show remarkable inductive effects (especially the two lower concentration treatments) on the invasion (**a**) and transformation (**b**) ability of HepG2 cells after 8-day irradiation to fluorescent white light with an intensity of 60 μmol photons/m^2/s. By contrast, the > 3 kD fraction of i$_8$-CD [i$_8$-CD$_{>3kD}$, 12 (low), 60 (med), and 300 (high) mg carbon/L] and CDs without irradiation [n-CD, 12 (low), 60 (med), and 300 (high) mg carbon/L] show no obvious effects on the invasion (**a**) and transformation (**b**) ability of HepG2 cells. The dark grey dashed lines show the value of the control treatment without any addition of CDs while the pink dashed lines indicate the value of the positive control in the presence of 4 μM benzopyrene with well-known carcinogenic effects. Data are presented as the mean ± s.d. (n = 3 independent experiments). Source data are provided as a Source Data file.

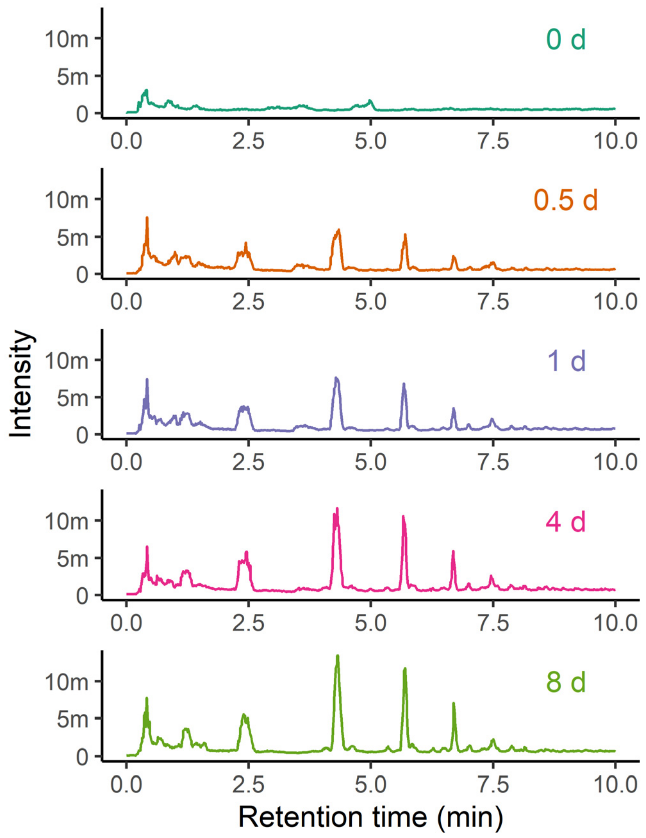

Fig. 6 | Total ion chromatograph of the degradation products of the laboratory-synthesized carbon dots (CDs) irradiated for 0, 0.5, 1, 4, and 8 days. The intensity of the total ion chromatograph increased, indicating the formation of a higher number or concentration of degradation products, when CDs were irradiated longer. Source data are provided as a Source Data file.

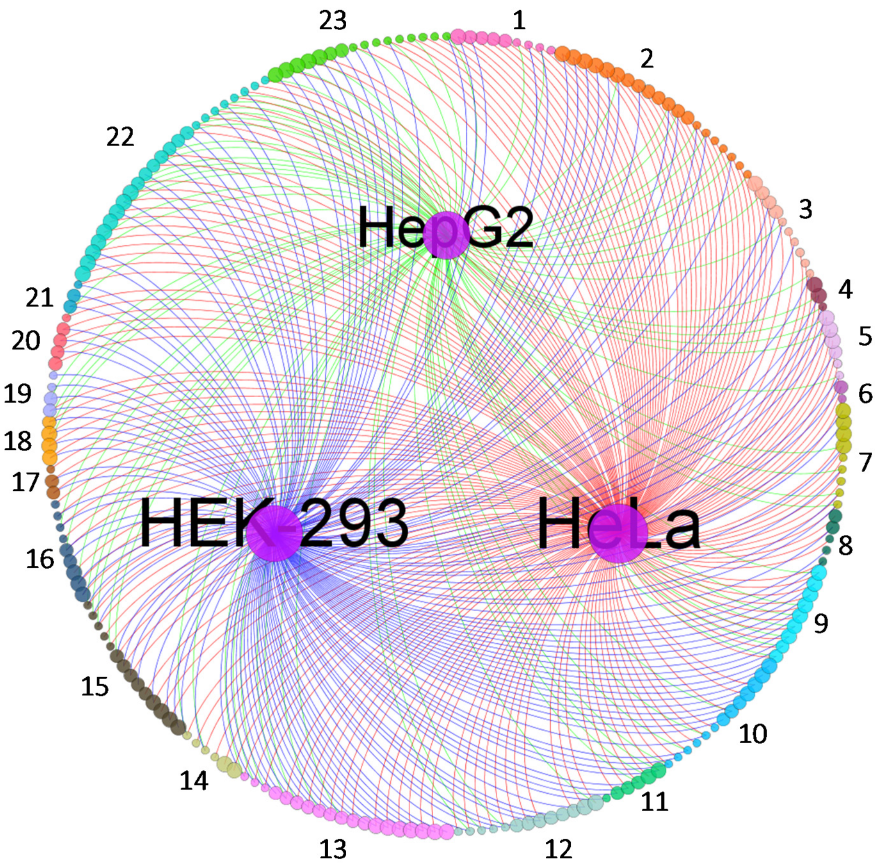

Fig. 7 | The network of the correlation between the cytotoxicity in each of the three cell lines and the formation kinetics of the photodegradation products with identified formulas or structures. The structures of 212 photodegradation products associated with cytotoxicity in at least one of the HepG2, HEK-293, and HeLa cell lines were identified and were divided into 23 groups according to their chemical structures. Each dot around the circle represents one substance and the degradation products in different groups are differentiated by different colors. The links between the dots of the photodegradation products and the cell lines (green – HepG2, blue – HEK-293, red – HeLa) indicate the correlation between the formation kinetics of the photodegradation products and the cytotoxicity in cells. The dot's size indicates the number of links. Source data are provided as a Source Data file.

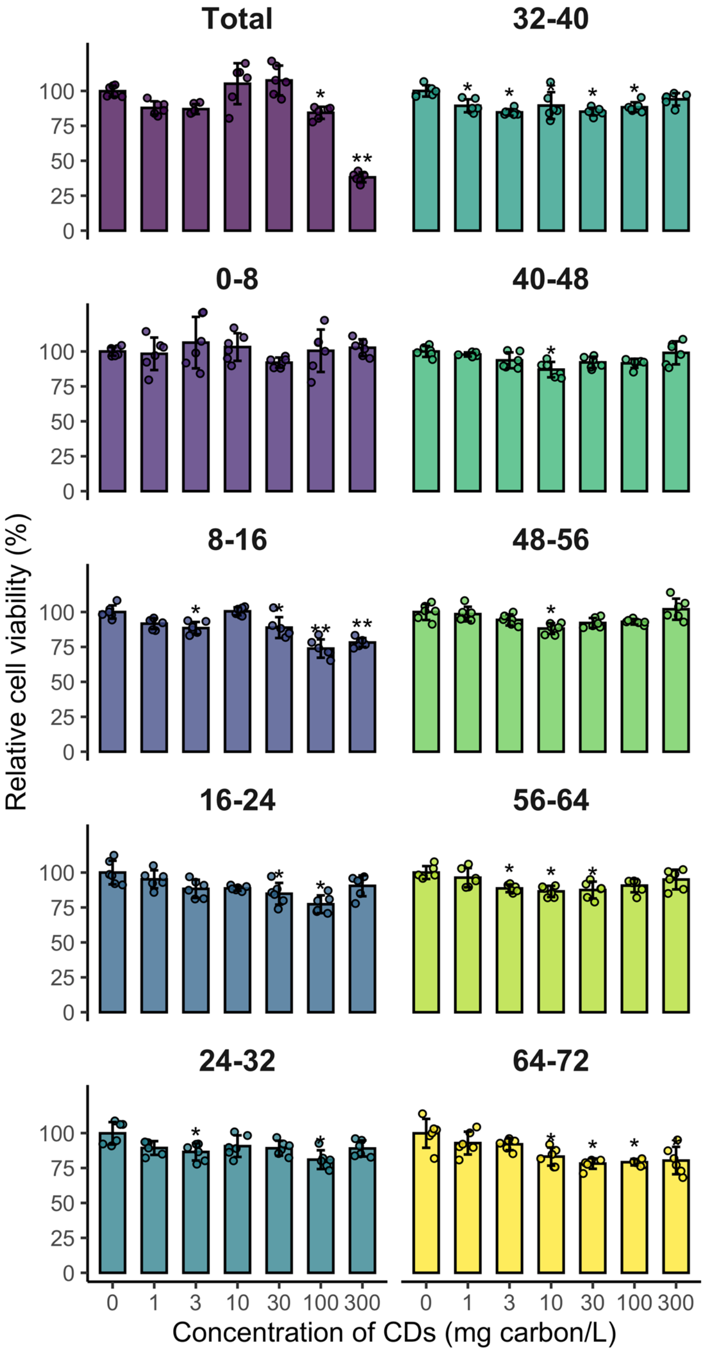

Fig. 8 | Cell viability testing using different fractions of the < 3 kD photolyzed products of laboratory-synthesized carbon dots (CDs). The total extract as a whole (i.e., sum of the 9 fractions) exhibited similar cytotoxicity to the raw photodegradation products, suggesting that most of the cytotoxic products were recovered by solid-phase extraction. Eight of the 9 fractions from the total extract show significant ($p < 0.05$, one-way ANOVA, see Source Data file for detailed *p* values) cytotoxicity to HepG2 cells, suggesting that the photo-induced cytotoxicity of the laboratory-synthesized CDs was a combined effect of the various

degradation products in the 8 fractions. The fractions were collected based on their different retention time (i.e., 0-8 min, 8-16 min, 16-24 min, 24-32 min, 32-40 min, 40-48 min, 48-56 min, 56-64 min, and 64-72 min) when the extract was eluted from the HPLC column. Significant inhibition was labelled by * or **. Data are presented as the mean ± s.d. (n = 6 independent experiments). Source data are provided as a Source Data file.

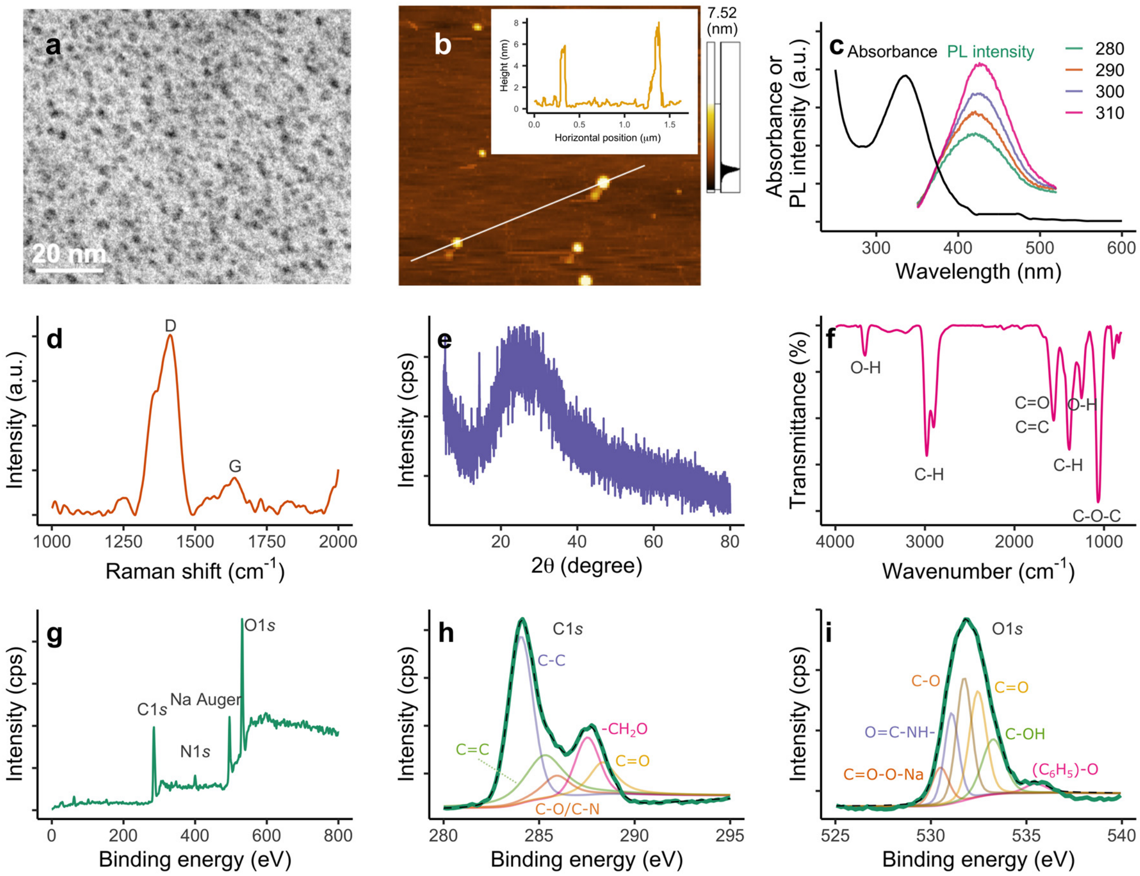

Fig. 9 | Physicochemical characterization of commercial carbon dots (CDs) from Sigma-Aldrich. a, Transmission electron microscopy (TEM) image shows CDs' average size is 4.6 nm. **b**, Atomic force microscopy (AFM) image shows CDs' average size is 4.9 nm. **c,** An absorption peak at 335 nm is seen in the UV-Vis spectrum (black curve). As excitation wavelength increased from 280 nm to 310 nm (in 10 nm increments), photoluminescence (PL) emission was red shifted from 421 nm to 426 nm. **d,** Raman spectrum of CDs showing the D and G band. I_D/I_G is 4.85, indicating commercial CDs have greater structural defects than laboratory-synthesized CDs. **e,** Broad peak at 25.6° in the X-ray diffraction spectrum confirms the structural disorder in the CDs. **f-g,** Fourier transform infrared (**f**) and X-ray photoelectron spectra (**g**) along with the deconvoluted C1*s* (**h**) and O1*s* (**i**) peaks reveal hydrophilic groups are present on the CD surface. Besides C and O, N is also present in commercial CDs as different from that of laboratory-synthesized CDs according to the results in (**g**). Images of more than 1000 particles of CDs were taken by TEM or AFM. Source data are provided as a Source Data file.

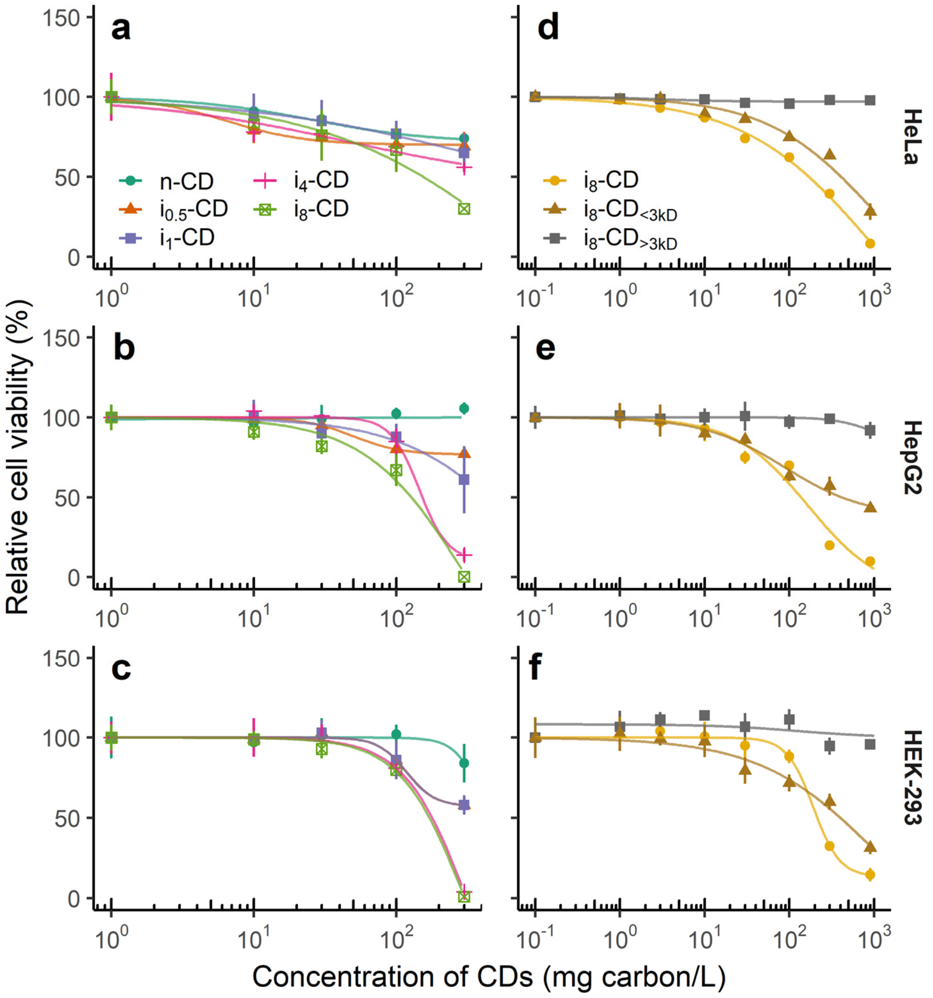

Fig. 10 | Cell viability testing of commercial carbon dots (CDs) from Sigma-Aldrich. a-c, Dose-response data show the cytotoxicity of CDs to HeLa (**a**), HepG2 (**b**), and HEK-293 (**c**) cells increased with irradiation time. The three cell lines were exposed to different concentrations (0, 10, 30, 100, 300 mg carbon/L) of CDs that have been irradiated with white fluorescent light (60 µmol photons/m^2/s) for 0 (n-CD), 0.5 ($i_{0.5}$-CD), 1 (i_1-CD), 4 (i_4-CD) and 8 (i_8-CD) days. **d-f,** Dose-response data show the photolyzed products in the < 3 kD fraction contributed substantially to the photo-induced cytotoxicity of CDs to HeLa (**d**), HepG2 (**e**), and HEK-293 (**f**) cells. The cytotoxicity of both the < 3 kD fraction containing photolyzed molecules (i_8-CD$_{<3kD}$) and the > 3 kD fraction containing degraded CDs (i_8-CD$_{>3kD}$) was compared with that of the total unfiltered suspension of i_8-CD at concentrations of 0, 1, 3, 10, 30, 100, 300, and 900 mg carbon/L. Data are presented as the mean ± s.d. (n = 6 independent experiments). Source Data are provided as a Source Data file.

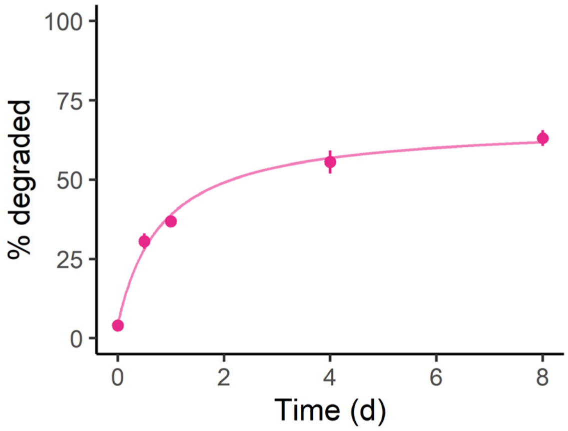

Fig. 11 | Photodegradation kinetics of the commercial carbon dots (CDs). Approximately 63% of the commercial CDs degraded into dissolved molecules < 3 kilo Daltons in size after 8 days of irradiation to white fluorescent light (60 µmol photons/m^2/s). Data are presented as the mean ± s.d. (n = 3 independent experiments). Source data are provided as a Source Data file.

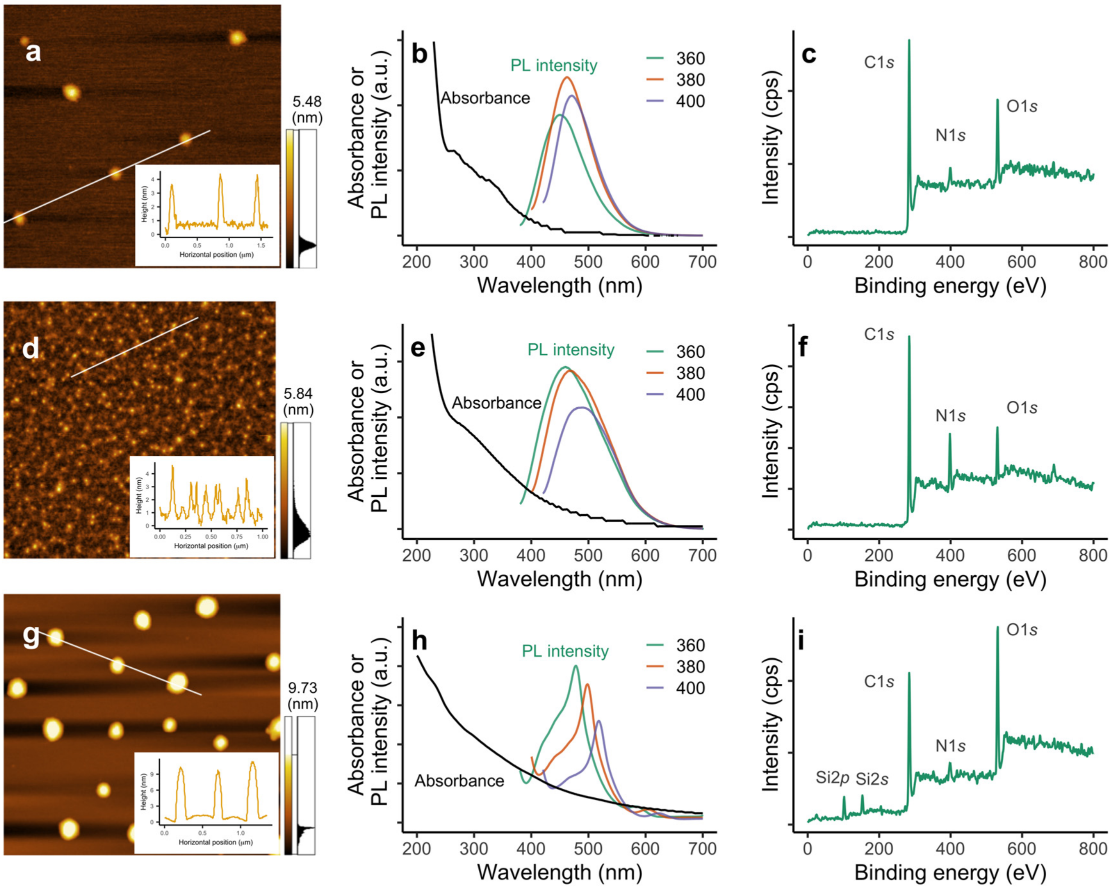

Fig. 12 | Physicochemical characterization of the three N- or Si-doped carbon dots (CDs). **a, d, g,** Atomic force microscopy (AFM) images show CDs' average size is 3.9 nm (**a**), 3.1 nm (**d**), and 9.4 nm (**g**) for the N-doped [CD_{N1} (**a**) and CD_{N2} (**d**)] and Si-doped [CD_{Si} (**g**)] CDs, respectively. **b, e, h,** No obvious absorption peak was seen in the UV-Vis spectrum (black curve) of CD_{N1} (**b**), CD_{N2} (**e**), and CD_{Si} (**h**). As excitation wavelength increased from 360 nm to 400 nm (in 20 nm increments), photoluminescence (PL) emission was red shifted from 449 nm to 471 nm for CD_{N1} (**b**), from 459 nm to 490 nm for CD_{N2} (**e**), and from 478 nm to 518 nm for CD_{Si} (**h**). **c, f, i,** X-ray photoelectron spectra reveal the existence of C, N, and O in CD_{N1} (**c**) and CD_{N2} (**f**) as well as the presence of C, N, O, and Si in CD_{Si} (**i**). Images of more than 1000 particles of CDs were taken by AFM. Source data are provided as a Source Data file.

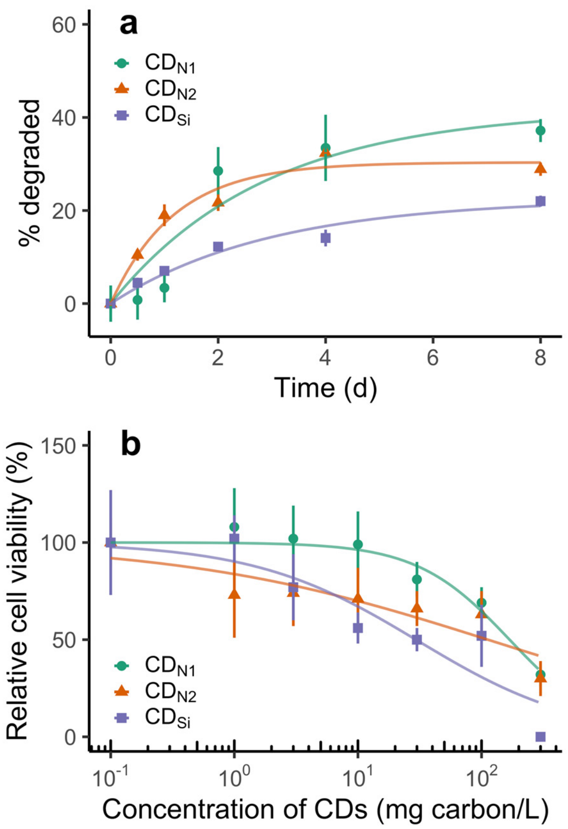

Fig. 13 | Photodegradation and cytotoxicity of the three N- or Si-doped carbon dots (CDs). **a,** Approximately 37.2%, 28.9%, and 22.0% of the N-doped (CD_{N1} and CD_{N2}) and Si-doped (CD_{Si}) CDs degraded into dissolved molecules < 3 kilo Daltons in size after 8 days of irradiation to white fluorescent light (60 μmol photons/m^2/s). **b,** Dose-response data show remarkable cytotoxicity of the < 3 kD photolyzed molecules from CD_{N1}, CD_{N2}, and CD_{Si} to HepG2 cells after 8-day irradiation. Data are presented as the mean ± s.d. (n = 3 independent experiments in **a** and n = 6 independent experiments in **b**). Source data are provided as a Source Data file.
