# Supplementary material for: Photodegradation of carbon dots cause cytotoxicity
Source: Nat Commun. 2021 Feb 5;12:812. doi: 10.1038/s41467-021-21080-z (PMC7864953; doi:10.1038/s41467-021-21080-z)
Supplement: Supplementary file 4 — Reporting Summary [file 41467_2021_21080_MOESM4_ESM.pdf]

Corresponding author(s): Si Wei, Xiao-Wei Zhang, Ai-Jun Miao

Last updated by author(s): Dec 10, 2020

Reporting Summary

Nature Research wishes to improve the reproducibility of the work that we publish. This form provides structure for consistency and transparency in reporting. For further information on Nature Research policies, see our Editorial Policies and the Editorial Policy Checklist.

Statistics

For all statistical analyses, confirm that the following items are present in the figure legend, table legend, main text, or Methods section.

n/a	Confirmed	
☐	☒	The exact sample size (n) for each experimental group/condition, given as a discrete number and unit of measurement
☐	☒	A statement on whether measurements were taken from distinct samples or whether the same sample was measured repeatedly
☐	☒	The statistical test(s) used AND whether they are one- or two-sided<br>*Only common tests should be described solely by name; describe more complex techniques in the Methods section.*
☒	☐	A description of all covariates tested
☐	☒	A description of any assumptions or corrections, such as tests of normality and adjustment for multiple comparisons
☐	☒	A full description of the statistical parameters including central tendency (e.g. means) or other basic estimates (e.g. regression coefficient) AND variation (e.g. standard deviation) or associated estimates of uncertainty (e.g. confidence intervals)
☐	☒	For null hypothesis testing, the test statistic (e.g. F, t, r) with confidence intervals, effect sizes, degrees of freedom and P value noted<br>*Give P values as exact values whenever suitable.*
☒	☐	For Bayesian analysis, information on the choice of priors and Markov chain Monte Carlo settings
☒	☐	For hierarchical and complex designs, identification of the appropriate level for tests and full reporting of outcomes
☒	☐	Estimates of effect sizes (e.g. Cohen's d, Pearson's r), indicating how they were calculated

Our web collection on statistics for biologists contains articles on many of the points above.

Software and code

Policy information about availability of computer code

Data collection	Analyst TF (1.5), R (3.6.1)
Data analysis	SPSS (23.0), Gephi (0.9.2), Matlab (R2016a), PeakView (1.2), R (3.6.1), Cytoscape (3.7.2), Xepr

For manuscripts utilizing custom algorithms or software that are central to the research but not yet described in published literature, software must be made available to editors and reviewers. We strongly encourage code deposition in a community repository (e.g. GitHub). See the Nature Research guidelines for submitting code & software for further information.

Data

Policy information about availability of data

All manuscripts must include a data availability statement. This statement should provide the following information, where applicable:
- Accession codes, unique identifiers, or web links for publicly available datasets
- A list of figures that have associated raw data
- A description of any restrictions on data availability

The data supporting the findings of this study are available within this article and its Supplementary Information files. All other relevant data are available from the corresponding authors upon reasonable request. The source data underlying Figs. 1–4, and Supplementary Figs. 1b–i, 2, 4, 5, 6, 7, 8, 9b–i, 10, 11, 12, and 13 are provided as a Source Data file provided with this paper.

Field-specific reporting

Please select the one below that is the best fit for your research. If you are not sure, read the appropriate sections before making your selection.

☐ Life sciences ☐ Behavioural & social sciences ☒ Ecological, evolutionary & environmental sciences

For a reference copy of the document with all sections, see nature.com/documents/nr-reporting-summary-flat.pdf

Ecological, evolutionary & environmental sciences study design

All studies must disclose on these points even when the disclosure is negative.

Study description	In order to answer whether CDs degrade under illumination and whether such photodegradation poses any cytotoxic effects, we performed the characterization of CDs, photodegradation kinetics experiments (n=3), cell viability assay (n=6), RHT experiment (seven 5-fold dilutions of CDs), and structure analysis of degradation products.
Research sample	Laboratory-synthesized and commercial CDs are both our research sample. We want to prove that photodegradation-induced cytotoxicity is common to CDs regardless of their chemical composition.
Sampling strategy	No sample size calculation was performed. Nevertheless, five types of CDs were chosen in the present study, which covered main categories of CDs including N-doped and Si-doped CDs. Therefore, the photodegradation-induced cytotoxicity observed in the present study is likely common to CDs.
Data collection	Y.Y.L. recorded data on CD characteristics by transmission electron microscope (JEOL JEM-2100), atomic force microscope (SPM-9700, Shimadzu, Kyoto, Japan), ultraviolet spectrophotometer (UV-2450; Shimadzu, Kyoto, Japan), fluorescence spectrophotometer (F-7000; Hitachi, Tokyo, Japan), X-ray diffractometer (ARL X'TRA; ThermoFisher Scientific, Massachusetts, USA), Fourier infrared spectrometer (Tensor27; Bruker, Massachusetts, America), Raman spectrometer (Labram HR800; Horiba Jobin-Yvon, Paris, France), X-ray electron spectrometer (PHI 5000 VersaProbe II; Ulvac-Phi, Kanagawa, Japan), dynamic light scattering particle sizer (ZetaPALS from Brookhaven Instruments, New York, USA, Malvern Nano-S90), their photodegradation kinetics by Elementar vario TOC analyzer (Elementar Analysensysteme GmbH, Germany), and cell viability by ThermoFisher Scientific Varioskan Flash multifunctional microplate reader (Infinite M200 PRO). N.Y.Y. recorded the high-resolution mass spectrometry data of CD photodegradation products by Triple TOF 5600. W.D.F. recorded the molecular biology data from the RHT experiment as well as from the cell invasion and transformation assays by Ion Torrent Proton and Synergy H4 Hybrid Reader. During data collection, several QC samples or control samples were detected to monitor the background and reproducibility of instrument and/or methods.
Timing and spatial scale	2016.11~2016.12, Lab-synthesized CD preparation and characterization as well as photodegradation; 2017.1~2017.7, Cell viability assay of lab-synthesized CDs at different degradation time points on the three cell strains; 2017.8~2017.12, Cell viability assay of the photodegradation products (< 3 kD) and CDs (> 3 kD) on the three cell strains with lab-synthesized CDs; 2018.1~2018.5, Cell viability assay of CDs from Sigma-Aldrich at different degradation time points on the three cell strains. 2018.6~2018.9, Cell viability assay of the photodegradation products (< 3 kD) and CDs (> 3 kD) on the three cell strains with CDs from Sigma-Aldrich. 2018.9~2018.12, RHT experiment; 2019.1~2019.3, Identification of the degradation products of lab-synthesized CDs; 2019.3.~2019.5, Identification of the degradation products of CDs from Sigma-Aldrich; 2019.11~2020.1 & 2020.6~2020.7, Photodegradation kinetics and the characterization of CDs from Sigma-Aldrich, cell viability assay of the total extract together with its 9 fractions of lab-synthesized CDs, Invasion and transformation of HepG2 cells exposed to lab-synthesized CDs; 2020.7, EPR experiment; 2020.8, Characterization, photodegradation and cell viability assay of another two N-doped and Si-doped CDs; 2020.11, CD degradation experiment in the dark.
Data exclusions	No data were excluded from the analyses.
Reproducibility	All attempts to repeat the experiment (either 3 or 6 independent experiments were repeated when necessary) were successful.
Randomization	Randomization is not relevant to our study. In the present study, we examined the photo-induced cytotoxicity of five CDs. The lab-synthesized CDs were systematically examined first. Their physicochemcial properties were characterized and their photodegradation kinetics, cytotoxicity, and photodegradation products were then examined. Afterward, another four CDs were tested to see whether the photo-induced cytotoxicity observed in lab-synthesized CDs can be extended to other CDs.
Blinding	As this is not a clinical research, we did not choose blinding during data acquisition in our study. Nevertheless, quality control of the data is ensured throughout the study. On one hand, several experiment (characterization of CDs, photodegradation kinetics of CDs and cell viability exposed by CDs photodegradation products) have been repeated (n=3-6), and the results show a good reproducibility. On the other hand, several QC samples or control samples were detected to monitor the background and reproducibility of instrument and/or methods during data collection, and the acquired data under a complete QA/QC system.

Did the study involve field work? ☐ Yes ☒ No

Reporting for specific materials, systems and methods

We require information from authors about some types of materials, experimental systems and methods used in many studies. Here, indicate whether each material, system or method listed is relevant to your study. If you are not sure if a list item applies to your research, read the appropriate section before selecting a response.

Materials & experimental systems

n/a	Involved in the study
☒	☐ Antibodies
☐	☒ Eukaryotic cell lines
☒	☐ Palaeontology and archaeology
☒	☐ Animals and other organisms
☒	☐ Human research participants
☒	☐ Clinical data
☒	☐ Dual use research of concern

Methods

n/a	Involved in the study
☒	☐ ChIP-seq
☒	☐ Flow cytometry
☒	☐ MRI-based neuroimaging

Eukaryotic cell lines

Policy information about cell lines

Cell line source(s)	All three cell lines (HepG2, HEK-293, and HeLa) used in the present study were bought from ATCC.
Authentication	All three cell lines (HepG2, HEK-293, and HeLa) were obtained with certificates from ATCC. None of the cells were authenticated by ourselves.
Mycoplasma contamination	The cell lines (HepG2, HEK-293, and HeLa) were not tested for mycoplasma contamination.
Commonly misidentified lines (See ICLAC register)	No commonly misidentified cell lines were used in the study.
